# Efficacy and toxicity of stereotactic body radiotherapy for un-resectable stage III non-small cell lung cancer patients unfit for concurrent chemoradiation therapy: a retrospective study

**DOI:** 10.1186/s13014-023-02333-1

**Published:** 2023-08-24

**Authors:** Zhen Jia, Fang Fang, Yangsen Cao, Xiaofei Zhu, XiaoYu Yang, Xueling Guo, Huojun Zhang

**Affiliations:** 1 Department of Radiation Oncology, Shanghai Changhai Hospital Affiliated to Navy Medical University, 168 Changhai Road, Shanghai, 200433 China; 2 Department of hepatic surgery, Shanghai Eastern Hepatobiliary Surgery Hospital, 255 Changhai Road, Shanghai, 200433 China

**Keywords:** Stage III non-small cell lung cancer, Stereotactic body radiotherapy, Chemoradiotherapy, Survival, Toxicity

## Abstract

**Background:**

In this study, we evaluated the efficacy and toxicity of stereotactic body radiotherapy (SBRT) as replacement strategy of conventionally fractionated radiation therapy in stage III non-small cell lung cancer (NSCLC) patients unfit for concurrent chemoradiation therapy (CRT).

**Methods:**

We analyzed the clinical outcomes in patients with unresectable stage III NSCLC who received SBRT from January 1, 2013 to December 31, 2018. Both induction and consolidation chemotherapy were allowed. The survival rates and toxicities were calculated using the Kaplan-Meier method, and potential risk factors were investigated by multivariate Cox regression.

**Results:**

A total of 213 consecutive patients who had received SBRT were enrolled. The median overall survival (OS) and progression-free survival (PFS) were 36.5 months and 16.1 months respectively. The estimated 1-, 2- and 3-year OS rates were 90.6%, 73.7% and 52.0%, respectively and the corresponding PFS rates were 69.5%, 25.4% and 15.0%, respectively. Treatment failures were largely (n = 151, 70.9%) distant metastases, with low rates of local (n = 74, 34.74%) and regional (n = 76, 35.68%) recurrences. In 13.1% patients (n = 28), ≥ grade (G) 3 toxicities were identified, including radiation pneumonia (n = 20, 9.4%) and bronchopulmonary hemorrhage (n = 8, 3.8%). None of the patients suffered from ≥ G 3 late toxic effects. Compared with patients with peripheral tumors, patients with central tumors had lower median OS (*P*<0.001) and the biological effective dose (BED) was not a predictor for OS.

**Conclusions:**

SBRT combined with chemotherapy for stage III NSCLC produced favorable treatment outcomes with acceptable toxicity. For patients with central tumors, an appropriate BED reduction can be considered. Further studies are warranted.

**Trial registration:**

Retrospectively registered.

**Supplementary Information:**

The online version contains supplementary material available at 10.1186/s13014-023-02333-1.

## Introduction

Non-small cell lung cancer (NSCLC) in stage III that cannot be resected accounts for about 20% of all patients diagnosed with NSCLC [1]. For these patients, the current standard treatment is concurrent radiotherapy and chemotherapy (CRT), followed by consolidation therapy with durvalmab [2–4]. In the real world in China, many patients with stage III NSCLC cannot afford CRT due to poor performance status and some comorbidities. For these patients, sequential CRT and exclusive radiation therapy (RT) may be alternative options. Historically, despite adequate treatment, overall survival rate at 5 years was 15–25% and locoregional relapse was 40–50% [5, 6].

In order to improve the clinical outcomes in this population, RTOG 0617 trial was conducted. The results showed that the survival rate of the high-dose group was inferior, and the median survival period was 20.3 months [7]. Based on the large volume of tumor burden or the number of metastatic lymph nodes (LN) in patients with stage III NSCLC, there is limited scope for increasing dose merely. A combination of several factors may explain the counterintuitive harmful effects of the higher radiation dose on the outcome in NSCLC, including dose escalation of 2 Gy/d and the prolongation of treatment time in the 74 Gy group [7]. Hence, dose escalation is not recommended in conventional fractionated concurrent CRT for stage III NSCLC. Hypofractionated RT is a kind of accelerated scheme which plays a significant role in clinical research, because of increasing biological effective dose (BED) without prolonging treatment time. Several reviews of stage III NSCLC radical-intent hypofractionated-RT found that there was a moderate linear relationship between local control (LC), overall survival rate (OS) and BED. It was concluded that radical-intent hypofractionated-RT with total treatment time ≤ 6 weeks was expected to be more beneficial than conventional radiotherapy with 2 Gy/Fx [8, 9].

Recently, the advanced radiotherapy technology has made the hypofractionated regimen more feasible. Stereotactic body radiotherapy (SBRT) includes a technology that can deliver higher dose and achieve higher BED through multiple radiation beams with steep dose gradient. At the same time, the new generation of machines including CyberKnife system allows accurate delivery of dose to the planned target volume (PTV), while protecting the surrounding healthy tissues [10]. Currently, SBRT has become the standard of care for patients with early-stage NSCLC who cannot be operated on. The 2–3year LC in patients with T1–2 disease was approximately 90% [11–13]. More recently, Yasuhiro et al. focused on CyberKnife-SBRT for Stage I peripheral NSCLC. The 2-year LC rate and PFS rate for patients with T1a/T1b and T1c/T2a disease was 100%, 90% and 95%, 65%, respectively [10]. The successful experience of SBRT in treating early cancer and the lessons learned from RTOG 0617 research will bring new insights into strategies to improve the effectiveness of local treatment [14].

Although, recently prospective phase II trials of SBRT in locally-advanced NSCLC have been published [15–17], the evidence of SBRT is greater in early-stage NSCLC [11, 18] and oligometastatic disease [19, 20]. Up to date, the role of SBRT is more important in the field of early primary NSCLC [11, 18] and oligometastatic disease [19, 20]. Some studies also had used SBRT as a boost after conventional RT for stage III NSCLC patients [21–23], but it is still unknown whether SBRT will be a safe and effective replacement of standard RT for unresectable stage III NSCLC. Therefore, the long-term results of patients with unresectable stage III NSCLC in our institution were analyzed retrospectively, with the aim of our study being was to explore the safety and effectiveness of SBRT combined with chemotherapy.

## Materials and methods

### Patients

This study has been approved by our institutional review board (IRB), and the informed consents were obtained from all patients. From January 1, 2013 to December 31, 2018, the database was queried to identify the consecutive patients with unresectable stage III NSCLC who were enrolled consecutively. Unresectable stage III patients unwilling or unfit to receive concurrent CRT (elderly age, serious baseline comorbidities, etc.) but suitable for RT should be assessed by the multidisciplinary team (MDT) which consisted of thoracic surgeons, medical oncologists and radiation oncologists and then the indications for SBRT were determined.

Eligible patients were ≥ 18 years with pathological or cytological confirmation of unresectable stage III NSCLC. All patients were staged on the basis of the 8th edition of the American Joint Committee on Cancer (AJCC) TNM classification. The tumor stage was evaluated with diagnostic positron emission tomography/computed tomography (CT). Clinical malignance was considered when LN with diameter ≥ 1 cm and definitive FDG uptake and if possible, LN metastasis was confirmed by pathology. MRI or CT imaging was previously necessary to eliminate patients with brain metastases. According to RTOG definition, primary central lung cancers were defined as lesions within 2 cm of the bronchial tree, esophagus, trachea, major vessels, brachial plexuse, heart or pericardium [24].

Other criteria include karnofsky performance status (KPS) of 70 or greater, and medical adaptability to induction and/or consolidation chemotherapy. Patients who have received chest irradiation, small-cell histology, distant metastasis (DM) or with disease not amenable to SBRT were unqualified. Patients with serious comorbidities, active interstitial lung diseases, or second primary cancer (with the exception of vesical superficial transitional cell cancer, cervical carcinoma in situ and cutaneous basal cell cancer) were forbidden for SBRT.

### Radiotherapy and chemotherapy

SBRT was delivered via CyberKnife® system (Accuray Incorporated, Sunnyvale, USA, G4). All treatment plans and target delineations were determined via computed tomography (CT)-based simulation with 1.5 mm slice thickness. The acquired CT images were transferred to a three-dimensional (3D) planning system (Accuray Incorporated, Sunnyvale, USA). At least two radiation oncologists worked together to delineate the targets, including primary lesions and positive LNs. The gross target volume (GTV) was defined as the gross disease determined in the imaging examinations. In patients receiving induction chemotherapy, the GTV were the residual disease defined on the basis of images after chemotherapy and pre-SBRT. Elective nodal irradiation was not conducted in this study. In most cases, the clinical target volume (CTV) was equaled to GTV. The PTV was generated based on a 5 mm margin expansion from CTV. If the tumor was adjacent to a critical organ, the expansion of CTV in this direction would be avoided.

The SBRT dose (range, 35–60 Gy) was administered in 5–10 fractions, which was calculated by the radiation oncologist on the basis of tumor location and size. Additionally, 95% of PTV should be covered by the prescription dose. The prescription isodose line was limited to 70–75%. The diameters of GTV ≤ 3 cm which were surrounded by lung parenchyma received 50–60 Gy in 5 fractions. The diameters of GTV greater than 3 cm or with chest wall contact received 35–60 Gy/6-7Fx. For GTV within 2 cm to the mediastinum or the brachial plexus, a dose of 35–60 Gy in 5–10 fractions was used. Dose constraints of organs at risk (OARs) like the spinal cord, heart, esophagus, normal lung, proximal bronchial tree, trachea, ipsilateral bronchus, were according to the American Association of Physicists in Medicine (AAPM) guidelines in TG-101. According to the clinical plan, dosimetric parameters were extracted from the dose volume histograms (DVHs) of contour lines.

The biologic equivalent dose with α/β = 10 Gy (BED_10_) of tumors was calculated to compare doses in different fractionations using the formula: BED = nd [1 + d/(α/β)] (n = fraction; d = dose per fraction).

Patients were allowed to receive a total of at least 4 cycles of combined chemotherapy, including induction and consolidation chemotherapy. Platinum-based doublet chemotherapy, including carboplatin and cisplatin, was administered. The chemotherapy regimen of squamous cell carcinoma and adenocarcinoma was mainly platinum-based with paclitaxel (cisplatin 75 mg/m^2^ on day 1, paclitaxel 200 mg/m^2^ on day 1, every 21 days), and platinum-based with pemetrexed (cisplatin 75 mg/m^2^ on day 1, pemetrexed 500 mg/m^2^, on day 1 every 21 days).The schedule of chemotherapy could be modified at clinical discretion. Since most patients were enrolled before the publication of the PACIFIC trial results, consolidation of immunotherapy was not required [4].

### Follow-up

In the first 2 years, the patients were followed up every 3 months and every 6 months after 2 years. The clinical examinations, supraclavicular ultrasound, blood tests, thoracic and abdominal CT examinations were necessary for each assessment. Both bone ECT scans and brain MRI were also performed annually.

Right after the treatment, acute toxicities were identified as occurring within 3 months and late toxicities occurred more than 3 months. Toxicities were graded depending on the National Cancer Institute’s Common Terminology Criteria for Adverse Events (CTCAE version 4.0).

Tumor response was evaluated according to Response Evaluation Criteria in Solid Tumors (RECIST) version 1.1. LC was achieved if the SBRT volume lacks treatment failure. Local-regional recurrence (LRR) was defined from the initial date of treatment to LRR (at the site of supraclavicular lymph nodes, mediastinal, hilar or primary tumor). The progression-free survival (PFS) was considered as the period from the initial treatment date to any treatment failure or death. OS was considered from the date of initial treatment to any cause of death or last follow-up.

### Statistical analysis

Statistics were performed using SPSS, version 25.0 s (IBM Corporation, Chicago, USA) and SAS software, version 9.4, for Windows (SAS Institute).

The LC was calculated based on competing risk. The PFS and OS were calculated using the Kaplan-Meier method, which generated the living curves. The difference in survival between groups was performed by the Log rank test. Patients who survived without failure were censored at the date of their last follow-up. Features of categorical and continuous variables and those of patients with peripheral or central tumors were compared using Fisher’s exact test and Wilcoxon’s rank sum test.

The univariate analyses were calculated by the Kaplan-Meier method. A univariate Cox proportional hazard model was to estimate hazard ratios (HRs) of the results in OS, LC, and PFS. Factors associated with *P* < 0.2 in univariate analysis was assessed using multivariate Cox proportional hazard analysis. A two-tailed *P*-value < 0.05 was considered statistically significant.

## Results

### Patients and treatment

During January 2013 and December 2018, a total of 273 Stage III NSCLC patients treated with SBRT were analyzed. Of these, 35 patients were excluded from treatment with surgical resection or radiotherapy prior to SBRT. 13 patients were excluded owing to the incomplete of SBRT. 12 patients missed the follow-up details. Ultimately, a total of 213 consecutive stage III NSCLC patients treated with SBRT were enrolled in this study (Table 1), in which 108 (50.7%) patients were with stage IIIA, 61 (28.6%) were with stage IIIB, and 44 (20.7%) were with stage IIIC. Two hundred of one patients (94.4%) had LN involvement consisted with N1 in 22 patients, N2 in 102 patients and N3 in 77 patients, respectively.

**Table 1:** **Pretreatment characteristics (N = 213).** KPS, karnofsky performance status; NOS, non-small cell lung cancer not otherwise specified; BED10, biologically effective dose; CT, chemotherapy; SBRT, stereotactic body radiotherapy

Variable	n	Percentage (%)
Age (yr)		
Md (range)	72(38–89)	
≤ 70	98	46.0
>70	115	54.0
Gender		
Male	175	17.8
Female	38	82.2
KPS		
90	71	33.3
80	125	58.7
70	17	8.0
History of smoking		
Yes	134	62.9
No	79	37.1
Primary pulmonary diseases		
Yes	85	39.9
No	128	60.1
T stage		
T1	36	16.9
T2	71	33.3
T3	49	23.0
T4	57	26.8
N stage		
N0	12	5.6
N1	22	10.3
N2	102	47.9
N3	77	36.2
TNM stage		
IIIa	108	50.7
IIIb	61	28.6
IIIc	44	20.7
Tumor diameter		
Md (range)	3.8(1.2–11.5)	
<4 cm	109	51.2
≥ 4 cm	104	48.8
Pathologic pattern		
Squamous cell carcinoma	93	43.7
Adenocarcinoma	109	51.2
NOS	11	5.2
Primary tumor lobe		
Left upper lobe	65	30.5
Left lower lobe	25	11.7
Right upper lobe	61	28.6
Right middle lobe	18	8.5
Right lower lobe		20.7
Primary Tumor Localization		
Central	86	40.4
Peripheral	127	59.6
BED_10_		
Md (range)	85.8(55–132)	
≤ 85.0 Gy	79	37.1
>85.0 Gy	134	62.9
Type of systemic therapy		
Induction CT + SBRT	107	50.2
Induction CT + SBRT + consolidation CT	19	8.9
Induction CT + SBRT + consolidation CT + immunotherapy	2	0.9
SBRT + consolidation CT	15	7.0
SBRT alone	70	32.9

In this study, most (n = 143, 67.1%) patients received chemotherapy. the majority (n = 107, 50.2%) of patients received platinum-based doublet induction chemotherapy as first-line treatment before SBRT; 19(8.9%) patients were treated with both induction chemotherapy and consolidation chemotherapy after SBRT, among them only two patients were enrolled in the study after September 2018, and subsequently received the consolidated immunotherapy of durvalumab; 15(7.0%) patients received consolidation chemotherapy and 70 (32.9%) patients received SBRT alone.

### Dosimetry data

All 213 patients completed the scheduled treatment in a median time of 5 days (range, 5–8 days). The median volume of GTV was 72.5cm^3^ (range: 5.2cm^3^-523.0cm^3^). The 70.0% ± 4.2 isodose lines were applied to cover the PTV. The median dose description was 48 Gy ± 6.6 Gy (range: 35.0–60.0 Gy) and the median fraction scheme was 6 ± 1.3 Fx (range: 5–10 times). In the meanwhile, the median BED_10_ range was 85.8 Gy ± 18.3 Gy (range: 52.73-132.0 Gy). The median dose description for both peripheral lung tumors and central lung tumors was 48.0 Gy. The median BED_10_ for peripheral lung tumors and central lung tumors were 90.0 Gy and 85.4 Gy, respectively (*P* = 0.002). The doses to the organs at risk (OARs) were illustrated in Additional file 1. The dose distribution of SBRT in different stage III was shown in Additional file 2.

#### OS and disease control

**Fig. 1:**
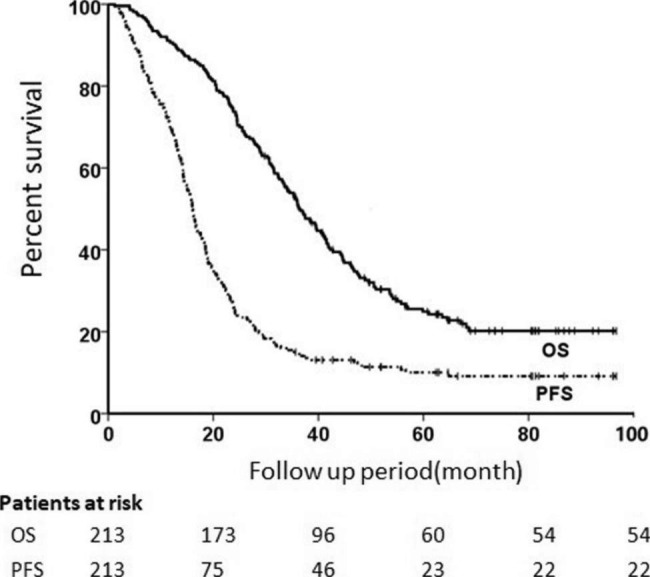
**OS and PFS of the total cohort.** OS, overall survival; PFS, progression-free survival

During a median follow-up of 40 (range 5.28–100.70) months, 54 (25.4%) patients were alive at last follow-up. Figure 1 illustrated the Kaplan-Meier survival analysis which showed that the median OS was 36.5 months (95%CI, 32.7 to 40.4) and the estimated 1-, 2-and 3-year OS rates were 90.6%±2.0%, 73.7%±3.0% and 52.0%±3.4%, respectively while the median PFS was 16.1 months (95%CI, 14.9 to 17.3) and the 1-, 2- and 3-year rates were 69.5%±3.2%, 25.4%±3.1% and 15.0%±2.5%, respectively. The results also demonstrated that disease progression was recorded in 89.7% of patients (n = 191). 1-, 2- and 3-year LC was 87.8% (95%CI 83.0-97.8%), 64.3% (95%CI 57.9-70.7%) and 57.2% (95%CI 50.7-64.0%).

**Fig. 2:**
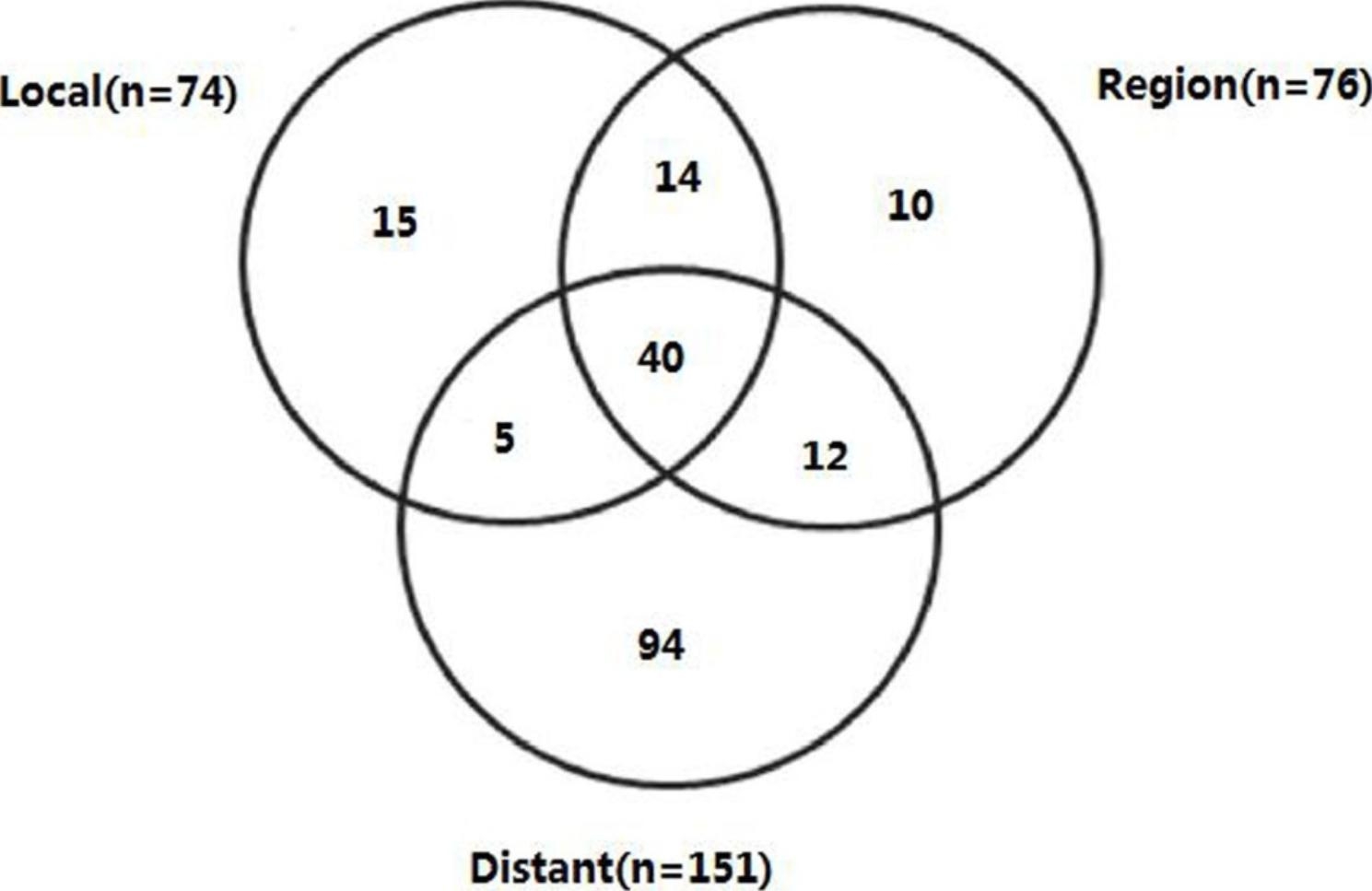
Pattern of treatment failure in total cohort

The patterns of treatment failure were analyzed in Fig. 2. Corresponding to 70.9% of all enrolled patients (151 of 213 patients), the dominant mode of treatment failure was distant. The overall incidence of local and regional recurrences was 34.74% (74 out of 213 patients) and 35.68% (76 out of 213 patients) respectively. Regional relapses and isolated local were uncommon with 4.69% (10 out of 213 patients) and 7.04% (15 out of 213 patients).

Results of the univariate Cox regression survival analysis were shown in Table 2. History of smoking (hazard ratio [HR], 1.394; 95% CI, 1.005–1.934), T stage (hazard ratio [HR], 1.678; 95% CI, 1.227–2.296), central or peripheral (HR, 0.535; 95% CI, 0.391–0.732), BED_10_ (HR, 0.625; 95% CI, 0.454–0.860) and pathologic patterns (HR, 1.652; 95% CI, 1.208–2.260) in favor of adenocarcinoma compared with others, were predictors for OS. TNM stage (hazard ratio [HR], 1.495; 95% CI, 1.119–1.98), T stage (hazard ratio [HR], 1.616; 95% CI, 1.215–2.149), central or peripheral (HR, 0.657; 95% CI, 0.429–0.877) and BED_10_ (HR, 0.626; 95% CI, 0.466–0.839) were predictors for PFS. TNM stage (hazard ratio [HR], 1.625; 95% CI, 1.101–2.481), T stage (hazard ratio [HR], 1.956; 95% CI, 1.299–2.944), central or peripheral (HR, 0.473; 95% CI, 0.316–0.707), BED_10_ (HR, 0.162; 95% CI, 0.104–0.251), pathologic pattern (HR, 1.530; 95% CI, 1.024–2.286), in favor of adenocarcinoma compared with others, were predictors for LC.

**Table 2:** **Univariate analysis of OS, PFS and LC for 213 patients with stage III NSCLC by SBRT combined with chemotherapy.** *Others includes squamous cell carcinoma and NOS. OS, overall survival; PFS, progression-free survival; LC, local control; NSCLC, non-small cell lung cancer; SBRT, stereotactic body radiotherapy; KPS, karnofsky performance status; NOS, non-small cell lung cancer not otherwise specified; BED_10_, biologically effective dose; PTV, planning target volume

Index	Median OS (month)	*p* value	Median PFS (month)	*p* value	Median LC (month)	*p* value
Age (yr)		0.417		0.406		0.698
≤ 70	37.4		16.0		58.8	
>70	36.0		16.1		55.3	
Gender		0.120		0.257		0.484
Male	35.3		15.6		56.2	
Female	46.6		20.1		60.8	
KPS		0.392		0.530		0.701
>80	39.6		16.9		58.3	
≤ 80	36.0		15.6		56.4	
History of smoking		**0.045**		0.056		0.292
Yes	33.1		14.3		55.0	
No	41.8		19.0		60.4	
Primary pulmonary diseases		0.722		0.261		0.207
Yes	36.1		16.7		60.8	
No	37.0		15.8		54.6	
TNM stage		0.166		**0.006**		**0.014**
IIIa	38.5		16.2		63.9	
IIIb + IIIc	36.1		15.9		49.7	
T stage		**0.001**		**0.001**		**0.001**
T1-2	44.2		18.6		65.4	
T3-4	32.3		13.0		47.8	
N stage		0.661		0.093		0.270
N0-2	34.0		15.6		60.4	
N3	37.2		17.6		50.1	
Pathologic pattern		**0.001**		0.071		0.037
Adenocarcinoma	44.6		18.6		62.2	
Others*	30.7		14.2		51.4	
Location of primary tumor		**<0.001**		**0.004**		**<0.001**
Central	26.9		13.9		45.9	
Peripheral	42.3		17.8		64.5	
BED_10_ (Gy)		**0.004**		**0.002**		**<0.001**
≤ 85.0	31.6		13.1		26.5	
>85.0	40.9		18.3		76.2	
PTV(cm3)		**<0.001**		0.001		0.001
≤ 108.1	50.6		22.0		68.9	
>108.1	30.7		12.9		21.6	

**Fig. 3:**
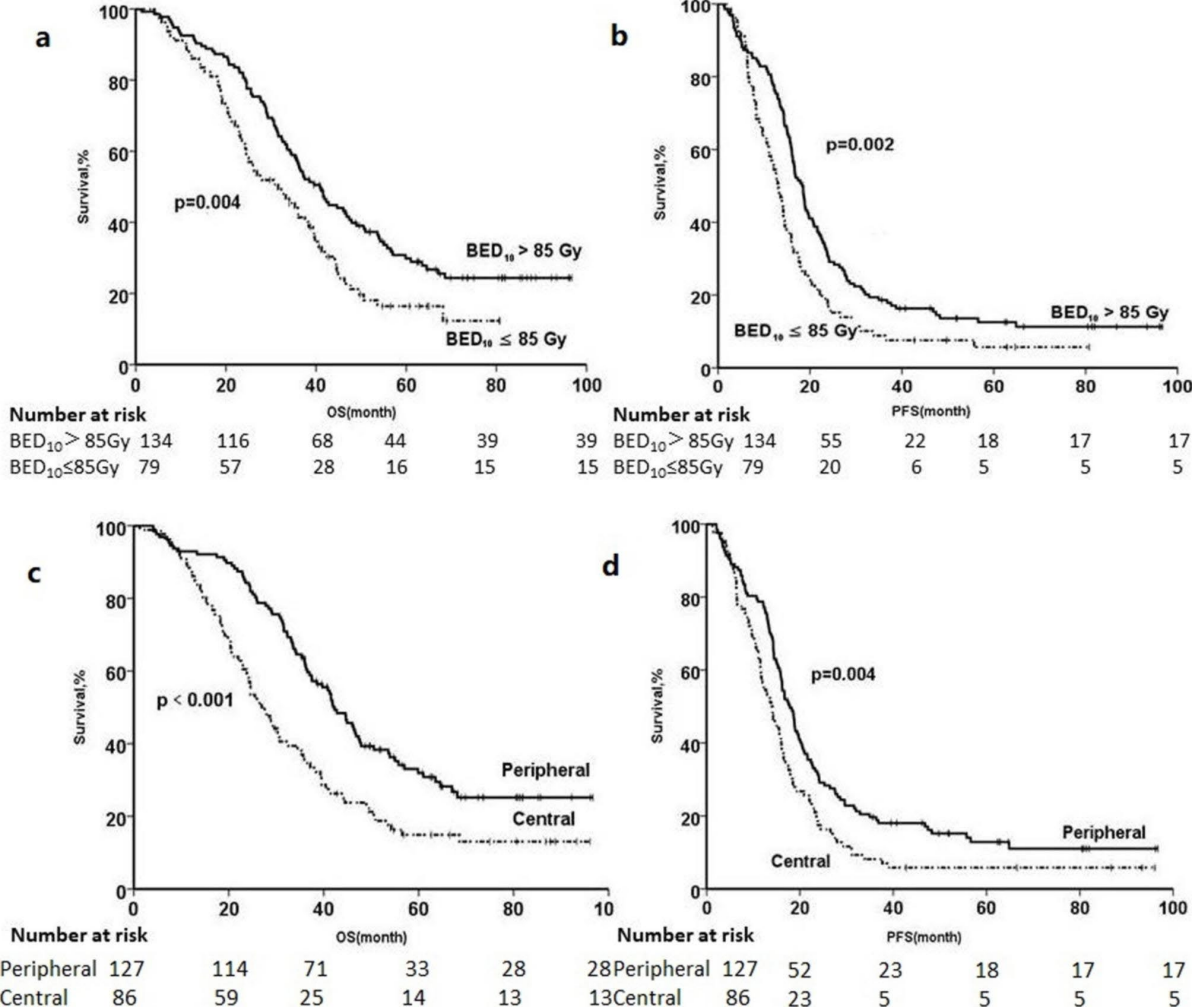
**(a) OS and (b) PFS curves of the BED**_**10**_ **> 85.8 Gy and the BED**_**10**_ **≤ 85.8 Gy; (c) OS and (d) PFS curves of the central NSCLC and peripheral NSCLC.** OS, overall survival; PFS, progression-free survival; BED_10_, biologically effective dose, α/β = 10 Gy

On the multivariate analysis, three parameters are independently correlated to OS so as to history of smoking no vs. yes (HR, 1.716; 95%CI, 1.077–2.733; *P* = 0.023), volume of PTV ≤ 108.1cm^3^ vs. >108.1 cm^3^ (HR, 2.479; 95%CI, 1.603–3.833; *P*<0.001), BED_10_>85.0 Gy vs. ≤85.0 Gy (HR, 0.576; 95% CI, 0.368–0.873; *P* = 0.010), and central vs. peripheral (HR, 0.544; 95% CI, 0.368–0.873; *P* = 0.006) (Table 3). The survival curves of different BED_10_ groups and central or peripheral groups were shown in Fig. 3.

**Table 3:** **Multivariate analysis of OS, PFS and LC for 213 patients with stage III NSCLC by SBRT combined with chemotherapy.** OS, overall survival; PFS, progression-free survival; LC, local control; NSCLC, non-small cell lung cancer; SBRT, stereotactic body radiotherapy; ADC, adenocarcinoma;BED_10_, biologically effective dose; PTV, planning target volume

Index		OS			PFS			LC		
Variables	reference vs.	HR	95%CI	*p* value	HR	95%CI	*p* value	HR	95%CI	*p* value
History of smoking	no vs. yes	1.716	1.077–2.733	**0.023**	1.568	1.052–2.338	**0.027**	1.981	1.124–3.491	**0.018**
TNM stage	IIIA vs. IIIB-IIIC	1.442	0.942–2.206	0.092	1.849	1.238–2.762	**0.003**	1.894	1.084–3.308	**0.025**
T stage	T1-2 vs. T3-4	1.401	0.889–2.206	0.146	1.223	0.810–1.845	0.338	1.173	0.661–2.084	0.585
Pathologic pattern	ADC vs. non-ADC	1.306	0.841–2.030	0.235	1.160	0.777–1.732	0.468	1.230	0.706–2.142	0.466
Location of primary tumor	central vs. peripheral	0.544	0.368–0.873	**0.006**	0.890	0.587–1.350	0.584	0.936	0.525–1.670	0.823
BED_10_ (Gy)	≤ 85.0 vs. >85.0	0.576	0.368–0.873	**0.010**	0.578	0.394–0.850	**0.005**	0.141	0.077–0.258	**<0.001**
PTV(cm^3^)	≤ 108.1 vs. >108.1	2.479	1.603–3.833	**<0.001**	1.785	1.223–2.605	**0.003**	2.265	1.322–3.882	**0.003**

Of all, 86 (40.4%) primary tumors were located in the central area, while 127 (59.6%) tumors were in the peripheral area. The median OS for patients with central and peripheral tumor was 26.9 months (95% CI 22.6–31.2) and 42.3 months (95% CI 37.0-47.5) (*P*<0.001) respectively. On the multivariate analysis, we also found that peripheral vs. central (HR, 0.588; 95% CI, 0.422–0.819; *P* = 0.002) was the independent factor of OS. Compared with the peripheral tumor cohort, the central tumor one had the larger tumor diameters (4.2 cm vs. 3.4 cm, *P* = 0.006) and lower BED_10_ (85.4 Gy vs. 89.8 Gy, *P* = 0.002).

For the further assessment, central tumor patients were divided into two groups based on their BED_10_ value (BED_10_ ≤ 85.0 Gy vs. BED_10_>85.0 Gy). And BED_10_ value (HR, 0.675; 95% CI, 0.424–1.075) was found not to be a predictor for OS.

### Toxicity

The acute and late toxic effects were enumerated in Table 4. There were 28 patients (13.1%) with ≥ grade (G) 3 acute toxicities. Among them, 26 patients (92.9%) were male with a median age of 72. Seventeen patients (60.7%) had a history of smoking and 22 patients (78.6%) had primary pulmonary diseases. Half of them had central primary tumor and the median BED_10_ was 74.7 Gy (range, 52.5-102.6 Gy). Potential predictors, including PTV D_max_, Heart mean dose, Heart D_15cm_^3^, Esophagus mean dose, Esophagus D_5cm_^3^, Lung mean dose, Lung V_5_, Lung V_20_, associated with ≥ G3 acute toxicity were calculated. Univariate analysis showed that only Esophagus D_5cm_^3^ correlated with ≥ G3 acute radiation toxicities (OR, 9.625; 95% CI, 1.075–86.175; P = 0.043). No independent prognostic factor was found in the multivariate analysis. In the meanwhile, one patient (0.5%) died of acute radiation pneumonitis without evident disease progression. The patient was a 79-year-old male diagnosed with T3N2M0 squamous cell carcinoma with a history of smoking and primary pulmonary disease. He developed coughs and chest pains after SBRT, which were temporarily relieved after treatment. A week later, his symptoms worsened and he was diagnosed with radiation pneumonia by CT and died 75 days after SBRT.

**Table 4:** **Treatment-related toxicities for 213 patients with stage III NSCLC by SBRT [n (%)].** NSCLC, non-small cell lung cancer; SBRT, stereotactic body radiotherapy

Treatment-related toxicities	Grade I	Grade II	Grade III	Grade IV	Grade V
Acute					
Neutropenia	11(5.2)	0(0)	0(0)	0(0)	0(0)
Cough	18(8.5)	29(13.6)	0(0)	0(0)	0(0)
Fatigue	43(20.2)	11(5.2)	0(0)	0(0)	0(0)
Radiation oesophagitis	29(13.6)	7(3.3)	0(0)	0(0)	0(0)
Radiation pneumonia	0(0)	15(7.0)	15(7.0)	4(1.9)	1(0.5)
Bronchopulmonary hemorrhage	0(0)	0(0)	4(1.9)	4(1.9)	0(0)
**Late**					
Cough	8(3.8)	4(1.9)	0(0)	0(0)	0(0)
Radiation oesophagitis	4(1.9)	4(1.9)	0(0)	0(0)	0(0)

Whereas late toxicities were rare with 8 (3.8%) patients developed G1 cough and 4 (1.9%) developed G2 cough. Late G1 and G2 radiation esophagitis occurred in 4 (1.9%) patients. There were no ≥ G3 late toxic effects.

## Discussion

The innovation of this study was the application of SBRT for the primary cancer and involved mediastinal LNs, instead of conventionally fractionated RT for stage III NSCLC patients. To the best of our knowledge, our study could be the first large-scale retrospective clinical study of SBRT combined with chemotherapy for the treatment of unresectable stage III NSCLC. Although most patients in our study were treated before the PACIFIC era and only two patients received consolidative immunotherapy, after a median follow-up of 40 months, the median PFS and OS reached 16.1 months and 36.5 months, respectively, with only 13.1% patients had ≥ G 3 acute toxicities and no ≥ G 3 late toxicities.

The safety and efficacy of platinum- based concurrent CRT for stage ΙΙΙ NSCLC patients have been explored by some groups. In their studies, the 5-year survival rate was approximately 30% and the median OS was in the range of 20–30 months [25, 26]. A number of SBRT studies were carried out to solve the LC problem, and different strategies were used to maintain the toxicity at an acceptable level with mixed results [27]. So far, there are 5 published studies of SBRT for unresectable locally advanced NSCLC, including three phase II single arm studies [15–17, 28, 29]. The total prescription dose range was 25 to 50 Gy, with BED_10_ in the range of 37.5 to 100 Gy. The median follow-up was 9–38 months. Total LC and median OS were 47.1-100% and 12–55 months, respectively. Karam et al. [28] and Cong et al. [29] reported that SBRT alone was a relatively safe and convenient treatment option for ultra-central primary tumor patients with inoperable advanced stage NSCLC and elderly (median age 79 years, range 65–100 years). Parisi et al. [15] reported a prospective phase-II trial which enrolled 17 patients treated with SBRT using tomotherapy combined with chemotherapy. The LC reached 19.8 months (95% CI 9.7 – not reached) but 70% of patients experienced acute G4 neutropenia, 24% G4 leukopenia, 4% underwent death after chemotherapy. Late toxicities were represented by 24% G3 dyspnea. Later, Kubicek et al. [16] reported their single arm phase II clinical study with a LC of 100% in 22 stage II-III NSCLC patients at a median follow-up of 23 months. Recently, Arcidiacono et al. [17] also conducted a single arm phase 2 trial to assess LC and safety of SBRT in unresectable locally advanced NSCLC patients. 50 patients were enrolled, of which 54% received CRT and 14% received adjuvant durvalumab. Nineteen patients (38%) had suffered local recurrence at a median time of 13 months. The 1-year and 3-year OS rates were 94% and 72% respectively. There was no patients experienced ≥ G3 toxicity. The existing studies were extremely limited, the results needed to be further verified and most of the above studies enrolled patients with stage II, IV, postoperative and recurrent patients [16, 17, 28, 29]. We are the largest study dedicated to unresectable stage III NSCLC.

With regard to the failure mode, an intriguing exploratory study on the recurrence mode showed that durvalmab reduced the risk of distant and intrathoracic recurrence [30]. However, compared with the extrathoracic chamber, the main site of treatment failure was the thoracic compartment (80% and 15%, respectively, for the durvalumab group) [4]. With the control of improving system, frequent intrathoracic progress was observed, which indicated that there still existed a space to increase the locoregional control with the plans and techniques of optimizing radiation, together with optimal systemic treatment [31]. Our current study indicated that dominant treatment failure was distant, according to 70.9% (151 of 213 patients) of all treated patients. Isolated local and regional relapses were uncommon with 15 (7.04%) and 10 (4.69%) patients, respectively. Our clinical outcomes were approximately the same as the durvalumab arm in PACIFIC trial [4]. Considering of only two patients with the consolidated durvalumab immunotherapy and 67.1% patients with combination chemotherapy, we explored the advantages of SBRT with chemotherapy for the therapy of unresectable stage III NSCLC patients. Also, the radiation field of SBRT did not result in a higher incidence of distant metastasis or local area progression, as only the primary tumors and positive lymph nodes were included in the designed target, providing additional evidence in support of omitting selective nodal irradiation [32]. While it is difficult to compare our results with PACIFIC trial, we believe that SBRT may lead to a resemble PFS and loco-regional control.

The severe toxic side effects after RT were contradicted to the dose escalation and were taken as the essential factors for limiting dose escalation [7]. Therefore, the higher targeting accuracy of SBRT resulted in a lower dose to the critical structures and might lead to a lower risk of toxicity. The incidence of ≥ G 3 toxicity was lower than that with conventional fractionation RT. The RTOG 0617 study found that ≥ G 3 pulmonary events occurred in 20% vs. 19% for the 60 and 74 Gy cohorts, respectively; ≥ G 3 esophagitis was observed in 7% vs. 21% [7]. In contrast, no irreversible severe toxicity was observed in previous SBRT for unresectable stage III NSCLC patients [16, 17, 28, 29]. The incidence of ≥ G3 toxicity was 0–24%, of which Parisi et al. considered that G4 neutropenia and leucopenia were related to chemotherapy [15]. The toxicity was also acceptable in our study. Only 13.1% patients (n = 28) were found to have developed ≥ G 3 acute toxicities of which 9.4% patients suffered from ≥ G 3 acute radiation pneumonia and no ≥ G 3 radiation oesophagitis.

The toxicity grade of esophagitis and the heart V5 (the percentage of heart volume receiving ≥ 5 Gy) were significant predictors of mortality [7]. SBRT’ s toxicity was generally mild for peripheral stage III NSCLC [33]. Furthermore, a body of evidence had been gathered that SBRT for central tumors was feasible and well tolerated [17, 24, 29]. RTOG 0813 study applied approach of 60 Gy in 8 fractions for centrally located early-stage lung cancer, prioritizing OARs tolerance over PTV coverage. Also, if tumors’ size was large and multiple OARs were of a concern, it was reasonable to use a conservative approach of 60 Gy in 15 fractions prioritizing PTV coverage [24]. No ≥ G 3 adverse event was observed in Arcidiacono et al.’s trial [17]. They considered that the limited toxicity could be explained by administering the ablative dose to parts of the GTV that were not strictly adjacent to the OARs in order to protect high-risk OARs. Cong et al. [29] founded that even with moderate doses, doses to the OARs may exceed the normal constraints. Although the low dose region of lung V5 was higher, the dose regions of V15 and V20 in SBRT-treated patients seemed much smaller than in patients treated with conventional fractionation RT. There are 86 (40.4%) patients with centrally located tumors in our study. We used different schemas to sustain a balance between efficacy and safety. The median BED_10_ for peripheral tumors and central tumors were 90.0 Gy and 85.4 Gy, respectively. The use of a comparable lower dose and moderate fractional regime may explain the limited toxicity registered. Because the central tumors always had larger volume and higher toxicity, we suggested that patients with central tumors were treated by BED_10_ ≤ 85.0 Gy SBRT, which represents a conservative and safe approach.

There are still some limitations in our study. First, the heterogeneity of our sample inevitably increased due to more complicated patients and retrospective study. Second, most patients were enrolled before the PACIFIC study was published. In our study, only two patients received immunotherapy, which was possibly the largest limitation in terms of toxicity, OS and PFS outcomes. As we gain more insight into the immunomodulatory role of RT, the use of SBRT in the appropriate context could enhance antitumor immune responses [34]. Undoubtedly, it should take more studies on this issue; however, our study could indicate hints and information for the “SBRT + immunotherapy” treatment in unresectable stage III NSCLC patients.

## Conclusions

Our study has shown that SBRT as part of a combination modality for management of unresectable stage III NSCLC has achieved favorable clinical outcomes with less toxicity. SBRT has the potential to be an effective therapeutic for patients who refuse to undergo concurrent CRT or are medically inoperable. For patients with central tumours, appropriate BED reduction may be considered. Further studies are warranted.

### Electronic supplementary material

Below is the link to the electronic supplementary material.

Supplementary Material 1

Supplementary Material 2

## Data Availability

The data presented in this study are available on request from the corresponding author in an anonymized form after data privacy check. The data are not publicly available due to data privacy regulations.

## Declarations

Helsinki, and approved by the local Ethics Committee of Medical Faculty, Changhai Hospital Affiliated to Navy Medical University. Patient consent was waived as the data were only analyzed in an anonymized form.

## Abbreviations

SBRT: Stereotactic body radiotherapy
NSCLC: Non-small cell lung cancer
CRT: Chemoradiation therapy
OS: Overall survival
PFS: Progression-free survival
BED: Biological effective dose
RT: Radiation therapy
LN: Lymph nodes
LC: Local control
PTV: Planned target volume
MDT: Multidisciplinary team
AJCC: American Joint Committee on Cancer
CT: Computed tomography
DM: Distant metastasis
GTV: Gross target volume
CTV: Clinical target volume
OARs: Organs at risk
AAPM: American Association of Physicists in Medicine
DVHs: Dose volume histograms
LRR: Local-regional recurrence
HRs: Hazard ratios
