# Correction: Efficacy and toxicity of stereotactic body radiotherapy for un-resectable stage III non-small cell lung cancer patients unfit for concurrent chemoradiation therapy: a retrospective study

**DOI:** 10.1186/s13014-023-02343-z

**Published:** 2023-10-11

**Authors:** Zhen Jia, Fang Fang, Yangsen Cao, Xiaofei Zhu, XiaoYu Yang, Xueling Guo, Huojun Zhang

**Affiliations:** 1 Department of Radiation Oncology, Shanghai Changhai Hospital Affiliated to Navy Medical University, 168 Changhai Road, Shanghai, 200433 China; 2 Department of hepatic surgery, Shanghai Eastern Hepatobiliary Surgery Hospital, 255 Changhai Road, Shanghai, 200433 China

**Radiation Oncology (2023) 18:140**

10.1186/s13014-023-02333-1

After publication of this article [1], the authors reported that in this article Zhen Jia and Fang Fang should have been denoted as equally contributing authors.

The original article [1] has been corrected.
