# Supplementary material for: Efficacy and toxicity of stereotactic body radiotherapy for un-resectable stage III non-small cell lung cancer patients unfit for concurrent chemoradiation therapy: a retrospective study
Source: Radiat Oncol. 2023 Aug 24;18:140. doi: 10.1186/s13014-023-02333-1 (PMC10463766; doi:10.1186/s13014-023-02333-1)

**Additional file 2. Axial, sagittal and coronal isodose distribution with SBRT of 45.0 Gy in 5 fractions for unresectable stage III NSCLC.** A. A IIIA stage (T4N0M0) patient. B. A IIIB stage (T3N2M0) patient. C. A IIIC stage (T4N3M0) patient. SBRT, stereotactic body radiation therapy; NSCLC, non-small cell lung cancer.


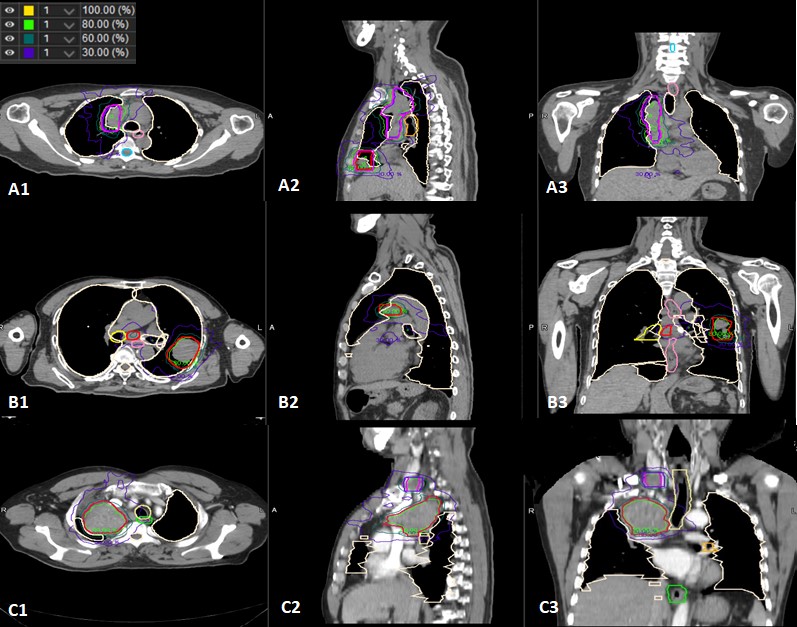

Supplement: Supplementary file 1 — Supplementary Material 1 [file 13014_2023_2333_MOESM1_ESM.docx]
