# Supplementary material for: Efficacy and toxicity of stereotactic body radiotherapy for un-resectable stage III non-small cell lung cancer patients unfit for concurrent chemoradiation therapy: a retrospective study
Source: Radiat Oncol. 2023 Aug 24;18:140. doi: 10.1186/s13014-023-02333-1 (PMC10463766; doi:10.1186/s13014-023-02333-1)
Supplement: Supplementary file 2 — Supplementary Material 2 [file 13014_2023_2333_MOESM2_ESM.docx]

**Additional file 1. Organs at risk for 213 Patients with stage III NSCLC by SBRT.** PTV, planning target volume; Dmax, maximum point dose; D_0.35cm3_, D_15cm3_, D_5cm3_, dose received 0.35cm^3^, 15 cm^3^, 5cm^3^ volume of organ at risk, respectively; Lung V20, volume of total lungs receiving 20 Gy or more.

| **Treatment Parameter** | **Mean(SD)** | **Range** |
| --- | --- | --- |
| PTV (Dmax), Gy | 54.10 (6.96) | 35.21-72.35 |
| Spinal cord (Dmax), Gy | 8.00 (4.19) | 1.23-33.00 |
| Spinal cord (D_0.35cm3_), Gy | 10.16 (4.03) | 1.54-24.32 |
| Heart mean dose, Gy | 4.28 (2.30) | 0.94-9.17 |
| Heart (D_15cm3_), Gy | 13.26 (7.01) | 1.32-31.16 |
| Esophagus mean dose, Gy | 4.68 (2.25) | 1.27-12.29 |
| Esophagus (D_5cm3_), Gy | 12.13 (16.43) | 2.44-21.03 |
| Lung mean dose, Gy | 6.95 (2.80) | 0.32-12.95 |
| Lung V20, % | 9.1 (3.2) | 6-28.0 |
